# Prescription distribution and inequities in diabetes care: A comparative analysis of continuous glucose monitoring access by diabetes status, ethnicity and socio‐economic factors in England

**DOI:** 10.1111/dme.70130

**Published:** 2025-09-03

**Authors:** Samuel Seidu, John Tetteh, Setor Kunutsor, Pratik Choudhary, Kamlesh Khunti, Ramzi A. Ajjan

**Affiliations:** ^1^ Leicester Real World Evidence Unit Leicester Diabetes Centre Leicester UK; ^2^ Leicester Diabetes Research Centre, University Hospital Leicester Leicester General Hospital Leicester UK; ^3^ Division of Musculoskeletal and Dermatological Sciences, School of Biological Sciences, Faculty of Biology, Medicine and Health The University of Manchester Manchester England UK; ^4^ Section of Cardiology, Department of Internal Medicine, Max Rady College of Medicine, Rady Faculty of Health Sciences, University of Manitoba Saint Boniface Hospital Winnipeg Manitoba Canada; ^5^ Clinical Population and Sciences Department, Leeds Institute of Cardiovascular and Metabolic Medicine University of Leeds Leeds UK

**Keywords:** continuous glucose monitoring, diabetes disparities, ethnicity, healthcare inequity

## Abstract

**Background:**

Diabetes affects over 3.3 million people in England, creating a significant health and economic burden. Continuous glucose monitoring (CGM) improves diabetes management but remains unevenly accessible, especially among Black and minority groups who face onset at younger ages, higher diabetes rates and complications. Updated NICE guidelines promote CGM access for all people with T1D and certain people with T2D, yet data on prescribing patterns in England are limited. This study investigates CGM prescribing across integrated care boards (ICBs) and primary care networks (PCNs), focusing on ethnicity and deprivation, to identify and address access disparities.

**Methods:**

Cross‐sectional analysis of publicly available data examined CGM prescribing patterns across England's PCNs, focusing on ethnicity and socio‐economic factors. Data from OpenPrescribing, the National Diabetes Audit and Public Health England were analysed through descriptive and inferential statistics, including regression and Intraclass Correlation Coefficient (ICC) calculations, to assess disparities in prescribing ratio per 1000 people.

**Results:**

Significant disparities in CGM prescribing across PCNs and ICBs are identified, shaped by ethnicity, age and socio‐economic factors. The mean items prescription ratio is 4.87 per 1000 people, ranging from 0.26 to 11.59. People with T1D are generally younger, with only 15.5% over 65, compared to 52.0% in T2D. White individuals represent 83.6% of T1D cases, while South Asians and Afro‐Caribbeans are more prevalent in T2D (14.5% and 5.3%, respectively). ICBs with below‐average CGM prescribing have a higher percentage of Afro‐Caribbean and South Asian populations compared to ICBs with above‐average prescribing. For T1D, Afro‐Caribbean representation is 6.7 (SD:7.0) in lower‐prescribing ICBs versus 2.1 (SD:2.8) in higher‐prescribing ICBs, and for T2D, it is 8.4 (10.4) versus 1.8 (SD:3.4) South Asian representation in low‐prescribing ICBs is 10.6 (SD:13.7) for T1D and 21.9 (SD:20.5) for T2D, compared to 3.2 (SD:4.9) for T1D and 6.5 (SD:9.7) for T2D in higher‐prescribing ICBs. CGM prescribing variance attributed to ethnicity and deprivation is 46.6% in T1D and 77.3% in T2D, indicating considerable socio‐demographic impact.

**Conclusion:**

This study reveals significant ethnic disparities in CGM access, with Afro‐Caribbean and South Asian groups facing a reduced prescribing ratio per 1000 people. Consistent NICE guideline adoption and targeted outreach are needed to improve equity in CGM access.

**What's new?**

**What is already known?**

Minority ethnic groups in England, particularly Afro‐Caribbean and South Asian populations, face unequal access to diabetes technologies like Continuous Glucose Monitoring (CGM), exacerbated by socio‐economic and healthcare inequalities.

**What this study has found?**

Significant CGM prescribing disparities exist, with Afro‐Caribbean and South Asian populations receiving fewer prescriptions despite a higher diabetes prevalence. NICE guideline adherence improves access.

**Implications of the study?**

Uniform NICE guideline implementation and culturally tailored outreach are essential to improving CGM access and outcomes for underserved populations.

## 1 BACKGROUND

Diabetes is a global health concern, with 537 million people affected worldwide, and this number is projected to reach 783 million by 2045.^1^ The prevalence of diabetes in England has also increased, with 3.34 million cases reported between 2017 and2018 and 2021 and 2022.^2^ Type 1 diabetes affects 270,935 people in England, while type 2 diabetes impacts 3,065,045 individuals.^2^ Diabetes significantly contributes to morbidity and mortality, causing 1.5 million deaths annually.^3^ In 2021/22, the UK's direct costs for diabetes were estimated at £10.7 billion, with slightly more than 40% attributed to diagnosis and treatment.^4^ Black and minority populations face a disproportionate impact, experiencing higher rates of diabetes and complications.^5^, ^6^

In recent years, continuous glucose monitoring (CGM) has emerged as a transformative technology for people with diabetes.^7^ CGM systems provide real‐time insights into glucose levels, allowing users to see patterns and trends that were previously difficult to detect.^8^, ^9^ This technology has been shown to significantly reduce the risk of hypoglycaemia, especially among older adults with type 1 diabetes.^10^ By offering continuous data, CGM helps individuals make more informed decisions about their diet, exercise and medication, leading to better overall glucose levels.^11^ These advancements have not only improved the quality of life for people with diabetes^12^ but also reduced acute complications associated with the condition.^13^

Emerging evidence highlights unequal access to CGM among individuals with T1D, particularly in the context of the U.S. healthcare system, which differs significantly from the NHS model. A study analysing trends from 2016 to 2022 revealed significant disparities in HbA1c levels and diabetes device use, stratified by race/ethnicity and insurance type. While the overall mean HbA1c improved from 8.7% to 8.4%, and CGM usage increased by 45%, these benefits were not equitably distributed.^14^ Non‐Hispanic Black individuals and those with public insurance experienced larger improvements in HbA1c (1.2%–1.6%), CGM use (30%) and other diabetes technologies compared to their non‐Hispanic White counterparts.^14^ These findings highlight persistent health inequities in this setting.^15^, ^16^, ^17^

The updated National Institute of Health and Care Excellence (NICE) guidelines advise that all adults and children with T1D should have access to CGM.^18^, ^19^ For individuals with type 2 diabetes on intensive insulin therapy (requiring two or more injections daily), CGM monitoring may be recommended in cases of frequent or severe hypoglycaemia, disabilities preventing finger‐prick testing or situations where they would otherwise need to test eight or more times a day.^20^ The NICE guidelines for type 2 diabetes specifically urge commissioners, providers and healthcare professionals to work towards equitable access to CGM. In particular, they recommend tracking CGM usage, identifying eligible groups with lower adoption rates, and developing outreach initiatives to support and inform these groups about CGM options and benefits.^20^

Reporting data on prescribing in England is essential for identifying and addressing disparities in access to critical medical treatments and technologies, including CGM. Equitable healthcare requires insight into which populations are receiving advancements in treatment, and which may be underserved. Data on prescribing ratio can highlight patterns and guide targeted efforts to reduce health inequalities across regions and demographics. Despite reports of unequal use of CGM in the United States,^14^, ^17^ which have informed policy reforms, no similar reports have been published in England. This absence of data limits opportunities to monitor and improve equitable access, making it challenging to ensure that all eligible individuals benefit from these essential medical resources. In England, NHS England delegates healthcare management to Integrated Care Boards (ICBs) within regional Integrated Care Systems (ICSs). General practices within each ICB collaborate through Primary Care Networks (PCNs) to deliver large‐scale, coordinated care tailored to community needs, enhancing patient outcomes and reducing health disparities. This study aims to report on CGM prescribing patterns across ICBs and Primary Care Networks PCNs in England, with particular attention to the role of ethnicity and socio‐economic deprivation on prescribing. By examining these factors, this work seeks to uncover potential disparities in access to CGM technology, providing insights that could guide efforts to promote equitable healthcare access for diverse populations throughout the country.

## 2 METHODS

This study employs a cross‐sectional data analysis, using publicly available data to examine CGM prescribing patterns across PCNs in England. A structured approach to analysing CGM prescribing, focusing on geographical and socio‐economic variables to identify disparities related to ethnicity and deprivation, was used.

### 2.1 Data sources

We used prescribing data in August 2024 from OpenPrescribing, focusing on GP practices and PCNs for “Detection Sensor Interstitial Fluid” per list size, analysed to assess CGM prescribing ratio per 1000 people.^21^ While prescribing patterns may vary slightly month to month, August is not typically affected by seasonal anomalies, such as winter pressures or Quality Outcomes Framework year‐end surges, and was therefore considered representative of stable prescribing behaviour. Additionally, since NICE recommended CGM for Type 2 Diabetes in June 2022, by August 2024, over 2 years had passed, allowing sufficient time for ICBs to adopt and implement the guidance. As such, prescribing in August 2024 reflects post‐implementation practice. We chose a cross‐sectional analysis at this time point to explore regional variation and provide a clear snapshot of CGM uptake across ICBs.

The OpenPrescribing data set is constructed from publicly available prescribing information provided by NHS England. It contains comprehensive details on medications prescribed by general practices across England and subsequently dispensed in community settings. This data set offers practice‐level insights, including the quantity and cost of prescribed medicines alongside their associated prescribing codes. Updated monthly, the data are curated by the OpenPrescribing team to support analysis of prescribing patterns and variations within the NHS primary care system. For this study, the data set was filtered, cleaned and processed to align with the research objectives. Core report data from the National Diabetes Audit, detailing registrations for Type 1 and Type 2 diabetes, along with demographic information on Index of Multiple Deprivation (IMD) and ethnicity^22^ was also used. Data from Public Health England Fingertips Data: IMD data specific to GP practices from the Department of Health and Social Care's Fingertips tool provided socio‐economic context.^23^ In this study, IMD 1 represents the most deprived quintile, while IMD 5 represents the least deprived quintile, reflecting socio‐economic variation across the population. We also used NHS Digital PCN to Sub‐ICB Mapping data from NHS Digital linking PCNs to their respective Sub‐ICBs to facilitate regional comparisons.^24^ Finally, we used NHSBSA former CCG to do Sub‐ICB mapping of data from NHSBSA, linking former CCGs to current Sub‐ICBs for comprehensive geographic analysis.^25^

### 2.2 Variables definitions

Primary Care Networks (PCNs) in England generally cater to populations ranging from 30,000 to 50,000 individuals. They promote collaboration among general practices to provide coordinated and integrated primary care services. Integrated Care Boards (ICBs) oversee the planning and allocation of NHS resources within their regions, aiming to enhance the integration of health and social care services. The focus of the analysis was on the three major ethnicities in England: White, south Asian and Afro‐Caribbeans. South Asian ethnicity here refers specifically to individuals of Indian, Pakistani or Bangladeshi descent, consistent with standard UK definitions. Afro‐Caribbean refers to Black African and Black Caribbean populations.

### 2.3 Statistical analysis

Our analysis included both descriptive and inferential approaches to thoroughly understand the disparity that exists in item prescription ratio per 1000 people by ethnicity and IMD. We estimated item prescription ratio using the formula below:

$$\frac{{\text{Items prescibed}}_{i}}{\text{Total number}\;{\text{registered population}}_{i}}\times 1000,$$

where *i* represent individual PCN.

Where necessary, we aligned ICB catchment areas with prescribing data using OpenPrescribing's organisational mapping to ensure consistency. This allowed us to calculate the prescribing ratio per 1000 registered patients in each ICB, enabling fair comparisons across regions regardless of population size. We conducted a descriptive analysis to summarise the item prescription ratio, using means and standard deviations. Since categorical variables were extracted as percentages reported across centres, we also summarised them using means and standard deviations. We assessed the normality of the data by visually inspecting histograms and normal probability plots, which provided an understanding of the data distribution and the suitability of parametric methods.

For PCN data, we employed inferential analysis to investigate the relationships between item prescription ratio per 1000 people and ethnicity as well as IMD factors. We used ordinary least squares (OLS) linear regression to estimate effect sizes, aiming to describe the relationship between the average number of item prescriptions per 1000 people and two key variables: the percentage of ethnicity and IMD within the PCN. The effect size from the OLS regression provided a quantitative measure of how strongly these factors were associated with prescription, helping us understand how variations in ethnicity and socio‐economic deprivation relate to prescription levels.

To delve further into the variation in prescription ratio per 1000 people and assess how much of it could be attributed to differences in ethnicity and IMD percentages, we calculated the Intraclass Correlation Coefficient (ICC). In this analysis, we aimed to determine how much of the variance in item prescription ratio per 1000 people was due to variations between different ethnic groups and IMD categories rather than within‐group variability.^26^ To estimate the ICC, we used a linear mixed effects model that incorporated random intercepts for each ethnicity and IMD status category, allowing us to partition the total variance into between‐group and within‐group components. Finally, we estimated the distribution of prescription items by diabetes status using the average percentage of individuals within each ethnic group diagnosed with a specific type of diabetes.

## 3 RESULTS

### 3.1 PCN level

In all, 1289 PCN data were extracted and the mean items prescription ratio per 1000 people was 4.9 (95% CI = 4.8–5.0) and it ranged from 0.26 to 11.59 (median = 4.8; IQR = 3.6–6.1). The proportion of patients aged 65 years and older differed significantly between T1D and T2D groups. In the T1D group, the mean percentage of people over 65 years of age was 15.5% (95% CI = 15.2–15.8), whereas in the T2D group, it was substantially higher at 52.0% (95% CI = 51.5–52.5) (Table 1).

**TABLE 1:** Descriptive summary from the PCN data.

Variable	Type 1 diabetes (*N* = 1289)	Type 2 diabetes (N = 1289)	Percentage missing
	Mean (SD)	Mean (SD)	
Patients aged 65+	15.5 (5.3)	52.0 (9.0)	3.1
Male sex	56.4 (4.0)	55.8 (2.1)	3.1
Ethnicity			
South Asian	7.0 (11.0)	14.4 (17.9)	2.9
Afro‐Caribbean	4.4 (5.8)	5.2 (8.5)	2.9
White	83.7 (16.9)	73.7 (23.6)	2.9
IMD			
IMD 1	19.5 (20.2)	21.2 (21.5)	3.1
IMD 2	20.1 (12.4)	21.0 (12.3)	3.1
IMD 3	20.4 (10.5)	20.3 (10.3)	3.1
IMD 4	20.2 (11.8)	19.5 (12.1)	3.1
IMD 5	20.3 (19.2)	18.1 (18.3)	3.1

Abbreviations: IMD, Index of Multiple Deprivation; PCN, primary care network; SD, standard deviation.

Ethnic composition varied between the two groups, with T1D showing a higher representation of White individuals with an average proportionc of 83.7 (SD 16.9) compared to 73.7 (SD 23.6) in T2D. Conversely, the mean percentage of Afro‐Caribbeans was least represented with a similar proportion across both groups, with 4.4 (SD 5.8) in T1D and 5.2 (SD 8.5) in T2D. For deprivation status, the T1D diabetes group had an IMD 1 mean proportion of 19.5 (SD 20.2) slightly lower than the 21.2 (SD 21.5) in T2D. For the IMD 5, T1D had a slightly higher mean percentage at 20.3 (SD 19.2) compared to 18.1 (SD 18.3) in T2D (Table 1).

**FIGURE 1:**
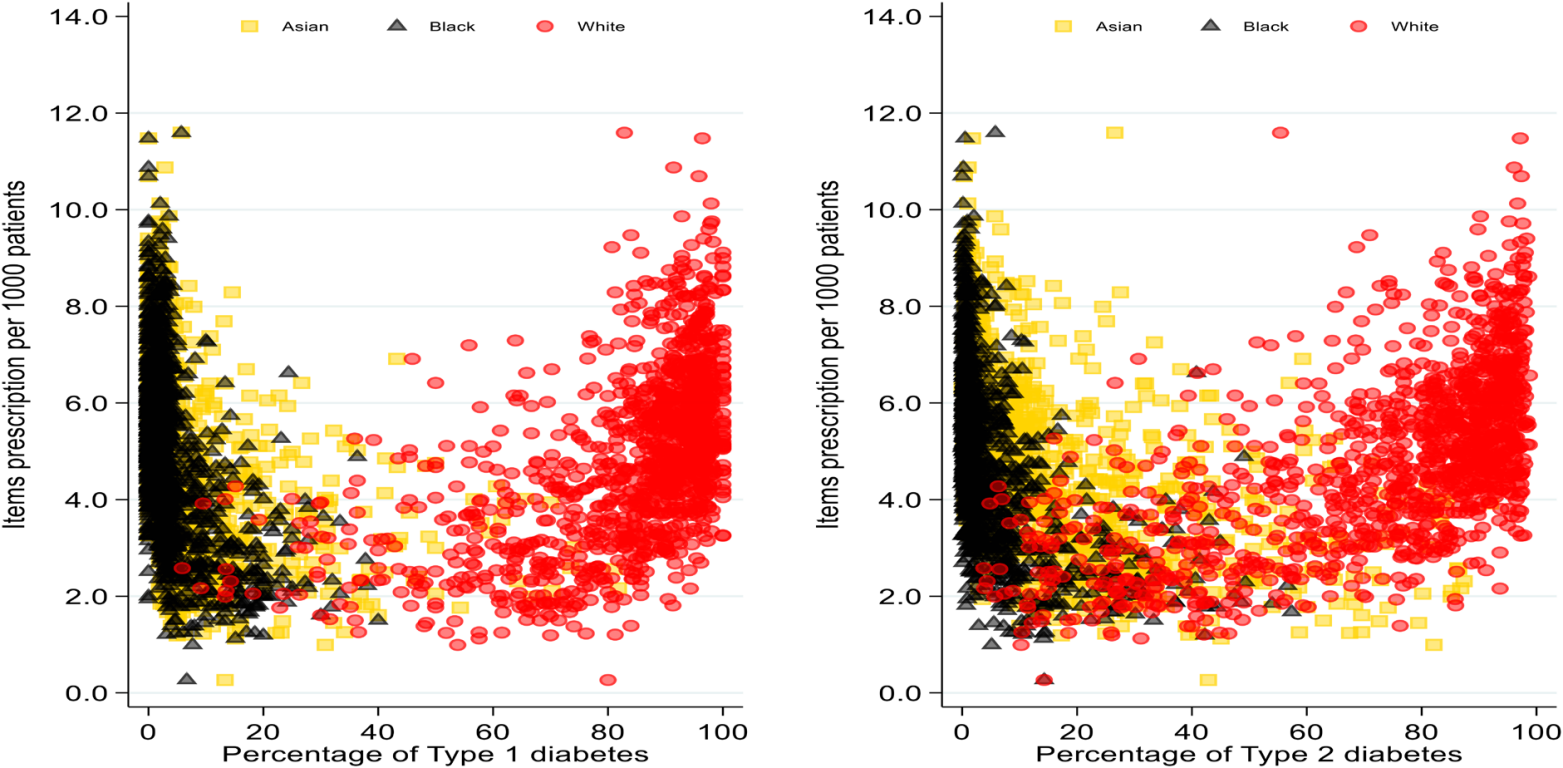
Distribution of items prescription per 1000 people by diabetes status. Red dots represent items prescription per 1000 people based on white population within PCN. Yellow dots represent items prescription per 1000 people based on south Asian population within PCN. Black dots represent items prescription per 1000 people based on Afro‐Caribbean population within PCN. The *y*‐axis showing the number of items prescribed per 1000 people and the *x*‐axis representing the average percentage of each diabetes type within ethnic groups. PCN, primary care network.

**FIGURE 2:**
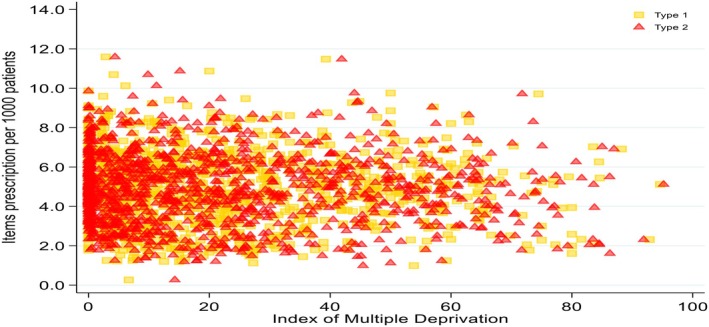
Distribution of items prescription per 1000 people by index of multiple deprivation status.

The distribution of items prescription per 1000 people by diabetes status is presented in Figure 1. In both T1D and T2D, people from White backgrounds had items prescription per 1000 people showing an upward trend. Areas with a larger proportion of White populations with diabetes have a higher prescription ratio per 1000 people. Afro‐Caribbean and South Asian populations generally showed a downward trend as the percentage of diabetes increased in these populations. The analysis showed that areas with a larger proportion of Afro‐Caribbean and South Asian populations had lower prescription. The distribution pattern indicates that regions with a higher percentage of White people with both T1D and T2D tend to have higher prescription item counts (Figures 1 and 2).

### 3.2 ICB level

**FIGURE 3:**
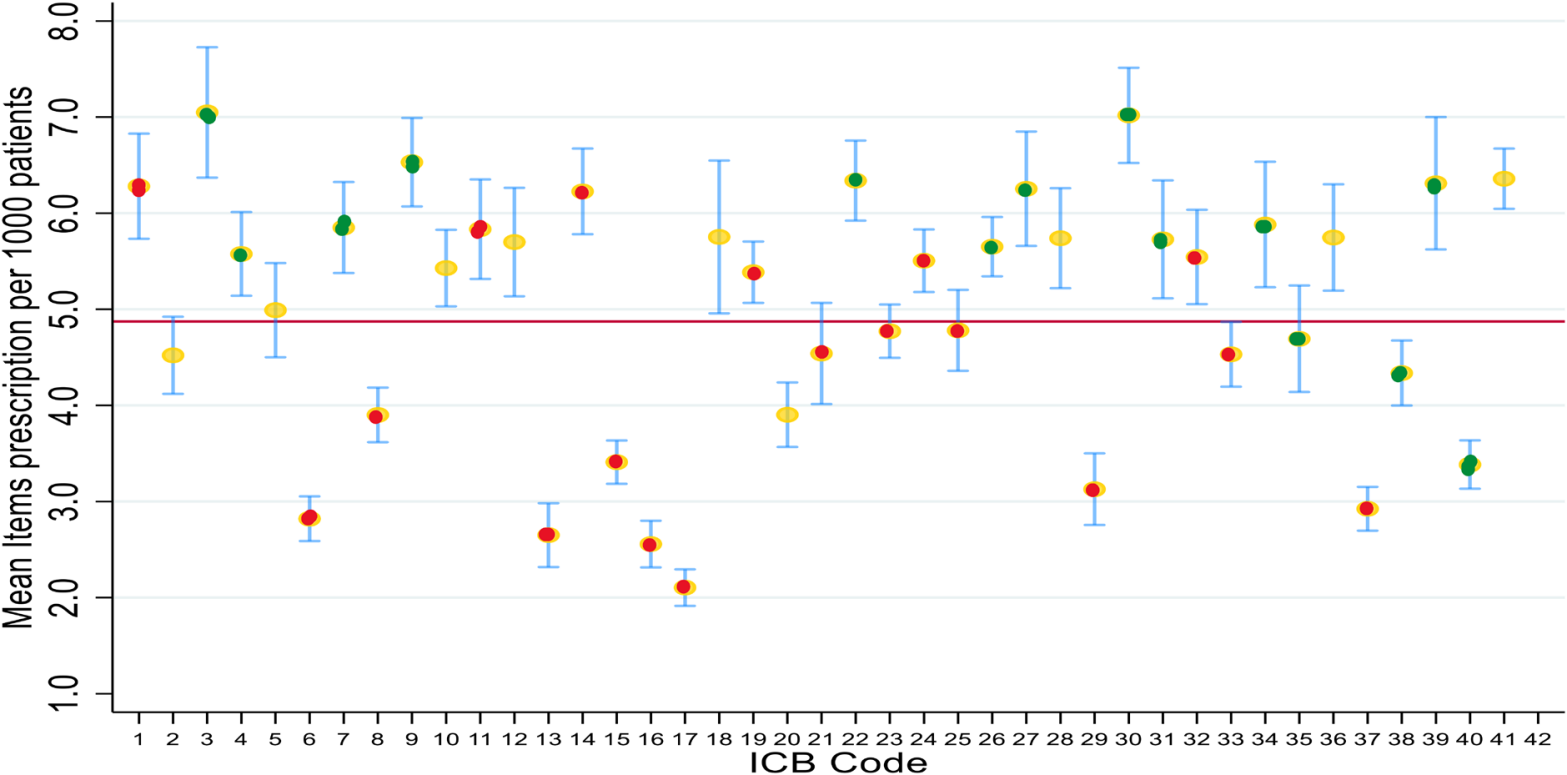
Distribution of items prescribed per 1000 by ICB sub region. Green dots: ICBs with full implementation of NICE guidelines and prescribing can be initiated in primary care. Yellow dots: ICBs with implementation of NICE guidelines and prescribing initiated in secondary care. Red dots: ICBs with no NICE guideline implementation. Red Horizontal bar represents overall average prescription rate across all ICBs. ICB, integrated care board; NICE, National Institute of Health and Care Excellence.

Compared to the overall average prescription ratio per 1000 people across all ICBs, 17 ICBs had prescription below this average, highlighting regional differences in prescription frequency. Among these 17 ICBs, majority (12 out of the 17) were areas with no NICE guidelines' implementation of CGM. Three regions were areas where there was implementation of NICE guideline prescribing with initiation allowed in primary care (full implementation), two are regions where prescribing initiation is done in secondary care (partial implementation) (Figure 3). Conversely, of the 24 ICBs with prescribing ratio per 1000 people more than the national average, majority of them (18 out of the 24) were in areas that implemented NICE guidelines for CGM prescribing. In 11 of these 18 ICBs, prescribing was initiated in primary care, with the remaining 7 ICBs only allowing initiation of CGM prescribing in specialist centres (Figure 3). Two ICBs had mean prescription rate above 7 (ICB 3 and 30) (Figure 3 and Appendix A). In six ICBs with no implementation of NICE guidance, the prescribing ratio per 1000 people with diabetes were still more than the national average, suggesting that clinicians are prescribing as per the guidelines irrespective of approval from their ICBs. Conversely in three ICBs with full implementation of NICE guidelines, the prescribing was still below the national average (Figure 3).

As shown in table 2, in both T1D and T2D, ICBs with below national average prescribing ratio per 1000 people of CGM have a higher percentage representation of Afro‐Caribbean and South Asian populations compared with ICBs with above average prescribing; Afro‐Caribbean 6.7 (SD 7.0) for T1D 8.4 (SD 10.4) for T2D compared with 2.1 (SD 2.8) for T1D and 1.8 (SD 3.4) for T2D, respectively. South Asian 10.6 (13.7) for T1D and 21.9 (SD 20.5) for T2D compared with 3.2 (SD 4.9) for T1D and 6.5 (SD 9.7) T2D, respectively. The percentage of people in the deprivation ranks was similar in the ICBs with below national average prescribing and those above national average prescribing in both T1D and T2D.

**TABLE 2:** Percentage of ethnic groups and IMD 1 ranks in ICBs below and above the national CGM prescribing ratio per 1000 people.

Variable	Below average (*N* = 659)		Above average (*N* = 630)	
	Type 1 diabetes	Type 2 diabetes	Type 1 diabetes	Type 2 diabetes
	Mean (SD)	Mean (SD)	Mean (SD)	Mean (SD)
Afro‐Caribbean	6.7 (7.0)	8.4 (10.4)	2.1 (2.8)	1.8 (3.4)
South Asian	10.6 (13.7)	21.9 (20.5)	3.2 (4.9)	6.5 (9.7)
IMD	19.3 (20.0)	21.5 (21.7)	19.6 (20.5)	20.8 (21.4)
IMD	21.2 (20.4)	18.3 (19.4)	19.3 (17.9)	17.7 (17.2)

Abbreviations: ICB, integrated care board; IMD, Index of Multiple Deprivation; SD, standard deviation.

**FIGURE 4:**
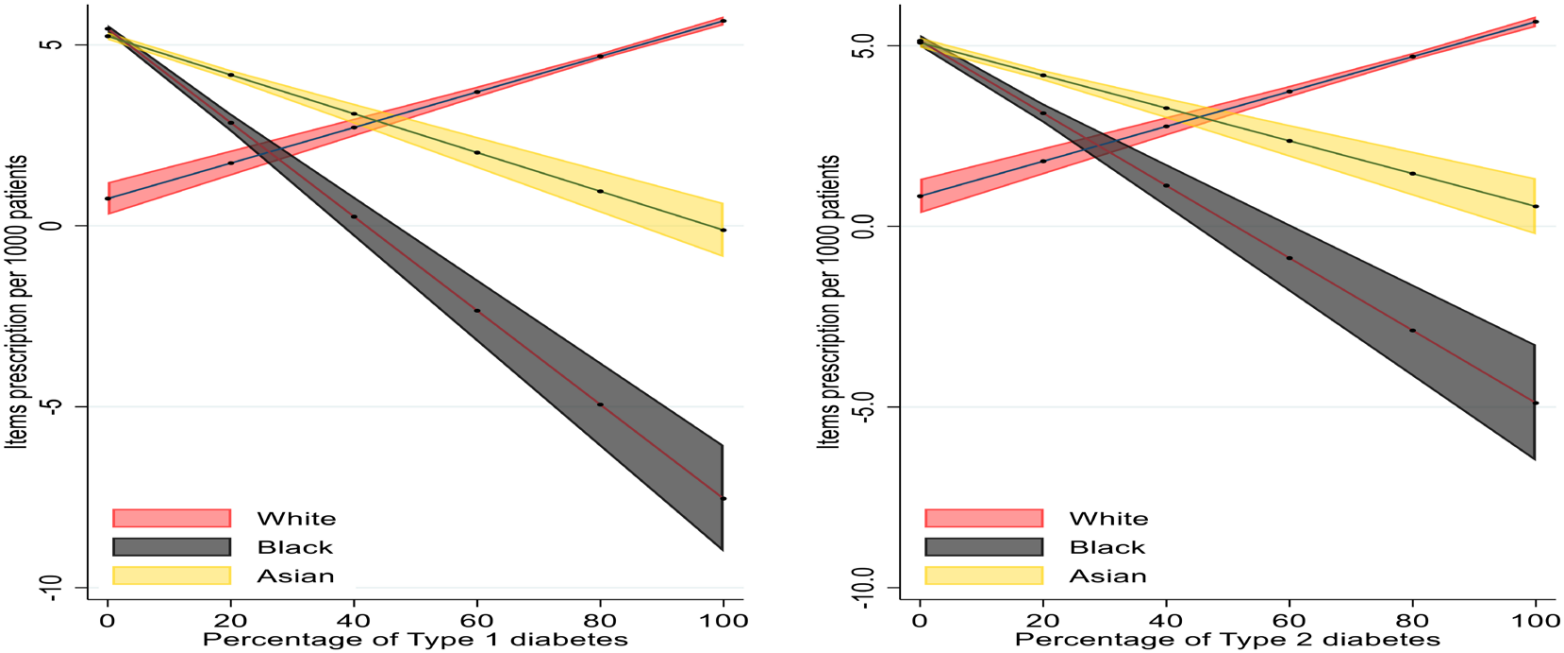
Predicted values of items prescribed by percentage of diabetes status.

The predicted values of prescription items by diabetes status are presented in Figure 4. The analysis showed a distinct trend in prescription ratio per 1000 people across racial groups as the percentage of T1D and T2D increases. For people of Afro‐Caribbean background with diabetes, the prescription ratio per 1000 people significantly declines with higher percentages of both Type 1 and Type 2 diabetes. People of South Asian ethnicity also showed a decline in the prescription ratio per 1000 people, though less pronounced than in the Afro‐Caribbean group. In contrast, people from White backgrounds exhibit a trend of increasing prescription ratio per 1000 people as the percentage of both diabetes types rises.

**FIGURE 5:**
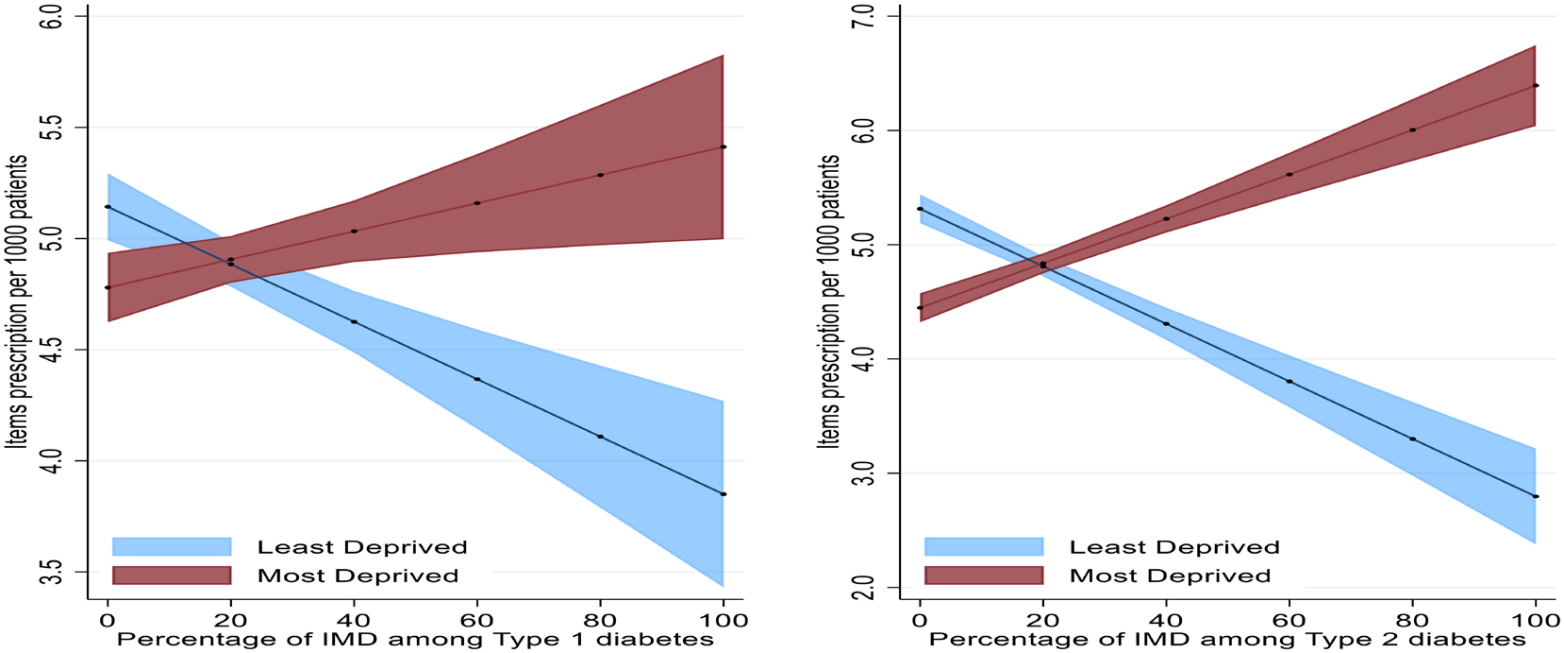
Predicted values of items prescribed by percentage of deprivation status.

Predicted values of items prescription ratio per 1000 people by percentage of IMD 1 and IM 5 are presented in Figure 5 below. Analysis showed that, for both T1D and T2D, prescription rates increase for the most deprived group as deprivation percentage rises, while they decrease for the least deprived group.

**FIGURE 6:**
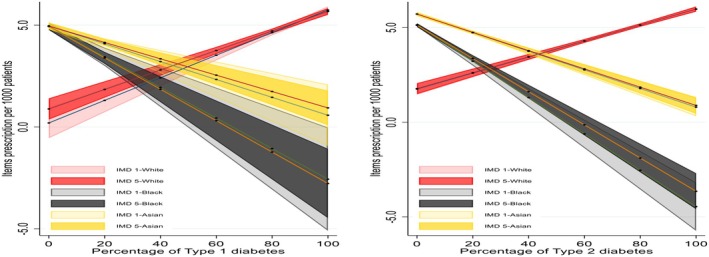
Predicted values of items prescription by ethnicity and deprivation interactions.

The predicted values of prescription items by ethnicity and deprivation interactions presented in Figure 6. Prescription ratio per 1000 people generally decreases for Afro‐Caribbean and south Asian groups as the percentage of T1D and T2D increases, regardless of deprivation level. People of White background showed an increase in prescribing regardless of deprivation. Afro‐Caribbean and south Asian populations tend to have lower prescriptions as diabetes prevalence increases, regardless of deprivation.

### 3.3 Contribution of ethnicity and deprivation on variations in prescribing ratio per 1000 people of continuous glucose monitoring

Ethnicity accounts for a moderate portion of the variance in items prescription ratio per 1000 people, with a slightly higher impact in T2D (ICC = 13.12%; 5.76–27.19) than T1D (ICC = 10.71%; 7.88–14.41). For IMD, analysis showed no meaningful effect of deprivation on prescription ratio per 1000 people. The combined effect of IMD within ethnicity shows the strongest influence on prescription rate variance, particularly for T2D. The ICC for T1D was 46.63% (43.26–50.05), while for T2D, it was 77.26% (68.83–83.94). These high ICC values suggest that the interaction between ethnicity and deprivation level was a major determinant of items prescription variability, especially in people with T2D (Table 3).

**TABLE 3:** Random intercept interclass correlation coefficients.

Random Intercept	Type 1 diabetes	Type 2 diabetes
	ICC [95% CI]	ICC [95% CI]
Ethnicity	10.7 [7.9, 14.4]	13.1 [5.8, 27.2]
IMD	2.6 [0.9, 7.1]	<0
IMD‐within‐Ethnicity	46.6 [43.3, 50.1]	77.3 [68.8, 83.9]

Abbreviations: CI, confidence interval; ICC, interclass correlation coefficient; IMD, Index of Multiple Deprivation.

## 4 DISCUSSION

Our results indicate that at the PCN level, there are significant trends in prescription ratios per 1000 people, influenced by age, ethnicity and deprivation status among people with T1D and T2D. Individuals with T1D tend to be younger, while those with T2D have a higher concentration in the over 65‐year‐old age group. White individuals comprise a larger proportion of those with T1D, while the T2D population has a greater representation of South Asian individuals. Afro‐Caribbean representation is largely similar across both groups. Prescription ratios per 1000 people are higher in areas with larger White populations, whereas regions with more South Asian or Afro‐Caribbean residents demonstrate lower prescribing ratios per 1000 people, indicating potential disparities in access to prescribed items across demographic and socio‐economic factors.

At the ICB level, CGM prescription ratio per 1000 people shows clear regional differences. Seventeen ICBs were below the national average, most lacking NICE guideline implementation. Among ICBs with above‐average prescriptions, the majority had implemented NICE guidelines, with many allowing CGM initiation in primary care or specialist centres. Interestingly, six ICBs without NICE guideline support exceeded the average rate, indicating clinician adherence to best practices in some areas, while three with full NICE implementation fell below the average.

ICBs with below‐average CGM prescribing ratio per 1000 people for both T1D and T2D show higher percentages of Afro‐Caribbean and South Asian populations compared to those with above‐average. Deprivation levels, however, are similar between ICBs with below‐ and above‐average prescribing. Prescription ratio per 1000 people shows a distinct trend by ethnicity: as T1D and T2D percentages increase, prescription ratios decrease significantly among Afro‐Caribbean and, to a lesser extent, South Asian populations, while White populations exhibit increasing prescription ratios.

The analysis shows distinct trends in CGM prescription ratio per 1000 people across ethnic and socio‐economic groups as diabetes prevalence increases. Among people of Afro‐Caribbean and South Asian backgrounds with T1D and T2D, prescription generally declines as the prevalence of diabetes rises, a trend more pronounced in Afro‐Caribbean groups. Conversely, White populations experience increasing prescription as diabetes prevalence grows. Additionally, prescription ratios per 1000 people rise for the most deprived groups as deprivation levels increase, while decreasing for the least deprived. However, the impact of deprivation was attenuated when intersected with ethnicity, with Afro‐Caribbean and South Asian populations with T1D and T2D showing declining prescription ratios as the prevalence of diabetes rises irrespective of deprivation status. White individuals consistently show higher prescription ratios regardless of deprivation level.

Ethnicity moderately affects CGM prescription ratio per 1000 people, with a slightly stronger impact in T2D. Although deprivation alone has little effect, when ethnicity and deprivation are combined, they strongly influence differences in prescription, especially in T2D. This suggests that both the ethnic background and socio‐economic status together play a key role in shaping how often CGM is prescribed.

The findings on CGM prescription, as influenced by ethnicity, age and socio‐economic status among people with T1D and T2D, align with and expand on patterns noted in existing literature. Studies have frequently shown that access to diabetes technologies like CGM is unevenly distributed across racial, ethnic and socio‐economic lines, with minority and lower‐income groups often at a disadvantage.^27^ This analysis is consistent with previous findings that White populations tend to have higher CGM adoption rates, while Afro‐Caribbean and South Asian groups encounter barriers, which may stem from differences in health service availability, healthcare access and socio‐economic disparities.^17^, ^28^, ^29^ Barriers to consistent technology use in children with type 1 diabetes, as identified in previous research, may have a greater impact on minority populations.^30^, ^31^, ^32^, ^33^ Additional challenges specific to CGM adoption in these populations may include healthcare provider biases, structural racism and broader social determinants of health.^34^ The interaction of ethnicity and deprivation as major determinants of CGM prescribing echoes findings that socio‐economic factors exacerbate healthcare inequalities, particularly for long‐term conditions such as diabetes.^28^ These results underscore the need for equitable access policies, a need supported by similar calls for reducing disparities in diabetes care access in recent studies.^35^ Studies indicate that White individuals with diabetes are more likely to receive CGM prescriptions compared to Afro‐Caribbean and South Asian populations, even when factors like socio‐economic status and healthcare needs are considered.^17^, ^29^ Such inequalities have implications for diabetes management outcomes, as CGM provides critical real‐time data that can enhance glucose control and reduce complications, particularly for those on intensive insulin therapy. The reduced access among Afro‐Caribbean and South Asian populations may exacerbate health disparities, leading to worse diabetes‐related outcomes and increased healthcare costs over time.^36^ Addressing these disparities through equitable prescribing policies and culturally sensitive healthcare practices could improve access to CGM technology, fostering better diabetes management across diverse populations and ultimately contributing to more equitable health outcomes.

Existing literature also emphasises the influence of age and diabetes type on CGM uptake, noting that older individuals, particularly those with type 2 diabetes, are less likely to use CGM. This may be due to clinical prioritisation, lifestyle factors, or limited technological literacy.^37^ The finding that some ICBs adhere to best practices despite not implementing NICE guidance may reflect the autonomy of local healthcare systems. It suggests that clinicians often apply evidence‐based approaches independently of formal mandates, a trend also observed in studies on healthcare access.^17^ The finding that ICBs with CGM prescribing ratios per 1000 patients above the national average are largely those that have implemented NICE guidelines reveals an important association between policy adherence and access to advanced diabetes management tools. This observation suggests that formal adoption of NICE guidance could play a key role in increasing CGM availability, potentially standardising care practices and promoting greater equity within regions that follow these guidelines. In contrast, the observation that ICBs with CGM prescribing rates below the national average are predominantly those that have not implemented NICE guidelines—and are often located in areas with higher proportions of Afro‐Caribbean and South Asian populations—raises concerns about inequitable access. This suggests a troubling association between reduced CGM provision and regions with greater ethnic minority representation. This pattern risks exacerbating existing health inequalities, as these groups, already facing barriers in healthcare access, may now encounter reduced access to CGM, a technology shown to improve diabetes control and outcomes.

Addressing this disparity requires concerted efforts to ensure that NICE guidelines are applied uniformly across all ICBs, paired with culturally informed outreach and support initiatives to improve CGM uptake among underserved communities. By promoting both adherence to guidelines and proactive outreach, healthcare systems could mitigate these inequalities, offering more inclusive and effective diabetes care for all demographics.

This study has several notable strengths and limitations. Among its strengths is the use of comprehensive aggregate data from sources like OpenPrescribing and the National Diabetes Audit, which lends reliability and breadth to the findings on CGM prescribing trends. The study's focus on socio‐demographic factors such as ethnicity, age and socio‐economic status provides valuable insights into potential disparities in healthcare access, identifying areas where policy intervention might be beneficial. Additionally, its analysis of NICE guideline implementation offers practical implications, showing that adherence to these guidelines is associated with improved CGM access, which can inform future healthcare policy decisions. However, as an observational study, it can only establish associations rather than causal relationships, limiting the ability to conclude definitively on the direct effects of factors like ethnicity or deprivation on prescribing. The use of aggregate data rather than individual‐level data also restricts the analysis's granularity, potentially obscuring specific patterns within subgroups. Furthermore, the study does not account for other confounding factors, such as regional healthcare funding variations or clinician attitudes, which could also influence the prescribing ratio per 1000 people, suggesting that some relevant variables may not have been fully considered. We did not adjust for diabetes prevalence within ICBs, as CGM is only prescribed for individuals with diabetes; regional prevalence differences are relatively modest and stable, and adjusting for them could introduce additional assumptions and obscure meaningful variation. However, we acknowledge this as a limitation and have explained our rationale in the discussion. Another limitation is the lack of linkage between prescribing data and outcomes such as HbA1c levels or hospital admissions, as data for other influential variables needed for such analysis were unavailable in the databases.

## 5 CONCLUSION

This study highlights significant disparities in CGM prescribing ratio per 1000 people, particularly influenced by ethnicity, with ICBs implementing NICE guidelines showing a higher prescribing ratio per 1000 people. The findings indicate that both ethnic background and deprivation level shape access to CGM, particularly disadvantaging Afro‐Caribbean and South Asian populations. To promote equitable diabetes care, consistent guideline implementation across all ICBs, combined with targeted outreach for underserved communities, is essential. Further research should explore individual‐level factors and address potential confounders impacting CGM access.

## AUTHOR CONTRIBUTIONS

All authors were involved in the design of the study. Samuel Seidu conducted the data screening and data collection. John Tetteh conducted the analysis. Setor Kunutsor double‐checked and supervised the analysis. Samuel Seidu wrote the first draft. All authors contributed to data interpretation, further drafting and critical revision of the manuscript for important intellectual content. Samuel Seidu is the guarantor of this work and, as such, had full access to all the data in the study and takes responsibility for the integrity of the data and the accuracy of the data analysis.

## FUNDING INFORMATION

KK and SS are supported by the National Institute for Health Research (NIHR), Applied Research Collaboration East Midlands (ARC EM), NIHR Global Research Centre for Multiple Long Term Conditions, NIHR Cross NIHR Collaboration for Multiple Long Term Conditions, NIHR Leicester Biomedical Research Centre (BRC) and the British Heart Foundation (BHF) Centre of Excellence.

## CONFLICT OF INTEREST STATEMENT

Samuel Seidu is in receipt of speaker honoraria from AstraZeneca, Boehringer Ingelheim, Janssen, Lilly, MSD, Abbott, Novo Nordisk, SB Communications, OmniaMed Communications, Roche, Napp Pharmaceuticals, NB Medical and Amgen; advisory board honoraria from AstraZeneca, Lilly, Boehringer Ingelheim, Janssen, Abbott, MSD, Novo Nordisk, Takeda and Sanofi; educational grants from Boehringer Ingelheim, Lilly, Novo Nordisk and Takeda and conference registration and subsistence from Boehringer Ingelheim, Janssen, Lilly, Novo Nordisk, Abbott and Takeda.

Ramzi A Ajjan: received honoraria for presentations and/or consultancy and/or research funding from Abbott Diabetes Care, AstraZeneca, Bayer, Boehringer Ingelheim, Bristol‐Meyers Squibb, Eli Lilly, GlaxoSmithKline, LifeScan, Menarini Pharmaceuticals, Merck‐Sharp & Dohme, NovoNordisk, Roche, Sanofi and Takeda.

Pratik Choudhary has received personal fees from Abbott, Dexcom, Insulet, Medtronic, Novo Nordisk, Lilly, Sanofi, Roche and Vertex and research support from Abbott, Dexcom, Medtronic and Novo Nordisk and is funded by an unrestricted educational grant from the European Foundation for the study of diabetes (EFSD) mentorship program supported by AstraZeneca.

Kamlesh Khunti has acted as a consultant and speaker for Novartis, Novo Nordisk, Sanofi‐Aventis, Lilly and Merck Sharp and Dohme. He has received grants in support of investigator and investigator‐initiated trials from Novartis, Novo Nordisk, Sanofi‐Aventis, Lilly, Pfizer, Boehringer Ingelheim and Merck Sharp and Dohme. KK has received funds for research, honoraria for speaking at meetings and has served on advisory boards for Lilly, Sanofi‐Aventis, Merck Sharp and Dohme and Novo Nordisk.

John Tetteh has no conflict of interest.

Setor Kunutsor has no conflict of interest.

## Data Availability

The corresponding author had full access to all the data in the study and takes responsibility for the integrity of the data and the accuracy of the data analysis.

## APPENDIX A Table 1

**Table:**

Number on Figure 3	Below average
1	N
2	Y
3	N
4	N
5	N
6	Y
7	N
8	Y
9	N
10	N
11	N
12	N
13	Y
14	N
15	Y
16	Y
17	Y
18	Y
19	N
20	Y
21	Y
22	N
23	N
24	N
25	Y
26	N
27	N
28	N
29	Y
30	N
31	N
32	N
33	Y
34	N
35	Y
36	N
37	Y
38	Y
39	N
40	Y
41	N

Abbreviations: N, no; Y, yes.

### A.1. Methods

We employed a contour‐enhanced funnel plot to visually assess asymmetry in the estimates of item prescriptions per 1000 patients across ICBs. To evaluate the degree of variability among the ICBs, we conducted a heterogeneity analysis using the *I* ^2^ statistic, which quantifies the proportion of total variation attributable to differences between ICBs rather than random chance.

Contour‐enhanced funnel plot of item prescription per 1000 patients.

Even though the funnel plot depicts asymmetry where ICBs with larger standard errors of item prescription per 1000 patients report larger effect size than the more precise ICBs, the contour‐enhanced funnel plot suggests that the estimates do not show any significant asymmetry (*p* > 0.1).
